# The Problem of Headache

**Published:** 1961-10

**Authors:** F. T. Page

**Affiliations:** Consultant Physician, Bristol Clinical Area


### THE PROBLEM OF HEADACHE

BY

F. T. PAGE, M.D., M.R.C.P.

*Consultant Physician, Bristol Clinical Area*

Headache is a symptom known and recorded throughout the ages. There is in the British Museum a papyrus dating from the twelfth century B.C. in which is depicted the exorcising of a half temple headache. Plato noted the relationship between headache and emotional disturbance. In America, headache is said to be the chief complaint in more than half the patients seeking medical advice and is common enough in this country in all fields of clinical activity. Headache may be the predominant symptom of the most malign and lethal conditions and yet is most commonly seen in the absence of organic disease. Many of the more serious conditions are no respectors of age. Intracranial tumours may, unlike most neoplasms elsewhere, occur in children or young adults. Intracranial infections, traumatic intracranial haemorrhage and vascular abnormalities also predominate in the younger age group. As a result, clinicians are always on guard lest they miss the early manifestations of organic intracranial disease. Unfortunately, the full investigation of a headache is a formidable procedure involving the services of an electro-encephalographic department, a skilled neuro-radiologist and a skilled neuro-surgeon. On the other hand, it may be said that, as a generalization, it is easier to diagnose the cause of a headache on "clinical" grounds than it is to diagnose the cause of almost any other common symptom.

### PAIN-SENSITIVE INTRACRANIAL STRUCTURES

Stimulation of the following structures can give rise to a sensation of pain:
(1) The major venous sinuses and their tributaries from the surface of the brain.
(2) The major arteries of the base of the brain.
(3) The dura in the anterior and posterior fossae.
(4) The meningeal arteries in the dura.
(5) The cranial and upper cervical nerves.
(6) The fifth, ninth, and tenth cranial nerves.

### CLASSIFICATION OF HEADACHE

A classification of the main causes of headache is set out in the accompanying table.

| | | |
|---|---|---|
| MUSCLE CONTRACTION | | |
| TENSION | | |
| PSYCHOGENIC | | |
| VASCULAR | Migraine | Histamine |
| | Fever | |
| | Hypertension | |
| | Temporal arteritis | |
| | Aneurysm | |
| | Intracranial haemorrhage | |
| | Intracerebral | |
| | Subarachnoid | |
| | Extradural | |
| | Subdural | |

| | |
|---|---|
| REFERRED | Ear, nose and throat<br>Eyes<br>Cervical spine<br>Teeth<br>Temporo-mandibular joint |
| EXPANDING LESIONS | |
| MENINGITIS | |
| CHANGES IN INTRACRANIAL HYDRODYNAMIC PRESSURE | High pressure<br>Low pressure |
| LOCAL | Paget's disease<br>Bone erosion, etc. |
| POST-TRAUMATIC | |
| NEURALGIAS | |

### *Headache and Fever*

Febrile infections frequently give rise to headache especially if the patient has septicaemia. For example, intense headache may be produced, in tropical countries, by malaria, typhoid and typhus fevers; and by influenza in more temperate climates. The headache is constant, generalized, dull and aching and of variable severity. Treatment of the underlying disease and simple analgesics will usually relieve such headaches. It is likely that this headache is the result of dilatation of intra-cranial arteries.

### *Headache and Hypertension*

This is a vexed problem. Since the two frequently co-exist it has sometimes been assumed that they constitute cause and effect. However, many hypertensives do not suffer from headache and it is a well-accepted fact that the level of blood pressure bears no relation to the presence, absence, or severity of headache. Many with mild hypertension have severe headache; many with severe hypertension have no headache. It is a general impression that headache is more common in those patients who are aware of their hypertension and certainly many hypertensive patients who suffer from headaches are those who might be expected to have headache even if normotensive.

Headache may be associated with hypertension in one of three ways. Firstly, in hypertensive crises, because of a sudden and intense rise in intravascular pressure; secondly because of an unstable cranio-vascular system such as is found in migraine; and thirdly as a result of nervous tension and anxiety. The last is the most common cause. Because of this, it is often more profitable to attempt to relieve emotional tensions than it is to prescribe the latest hypotensive drug, although such drugs may be required if hypertension enters the malignant phase.

### MIGRAINE

Migraine has been described as "A paroxsymal or episodic disorder characterized in its fully developed form by visual hallucinations, scotomas and other disturbances of cerebral function associated with unilateral headache and vomiting" (Brain, 1955). The term "hemicrania" was introduced by Galen (A.D. 131–201).

It is now well-established that the initial symptoms of migraine, that is, the visual hallucinations, scotomas, and other cortical symptoms and signs are caused by spasm of branches of the internal carotid artery and that the headache results from dilatation of extracranial arteries in the distribution of the external carotid. During the stage of headache, there may be visible dilatation of the superficial arteries and flushing of the face, while congestion of the conjunctival vessels and of the nasal mucosa may also be seen. If the headache is prolonged, the dilated vessels become oedematous and oedema may develop in the painful areas. It has been postulated, with experimental backing, that the oedema fluid contains a substance of low molecular weight which is responsible for lowering the pain threshold. Sustained contraction of the neck and scalp muscles may follow and may outlast the initial vascular pain.

It is often said that migraine tends to attack the intelligent and industrious members of society. Whether or not this is true there is little doubt that psychogenic factors are of greater importance than physical ones in the pathogenesis of migraine. Many patients exhibit such personality traits as over-conscientiousness, meticulousness and perfectionism. Some develop migraine when they can no longer suppress guilt or resentment or because they wish to remain in a state of dependence on others. By no means all patients with migraine have personality disorders but it is of great importance to recognize these when they exist.

### *Clinical Aspects of Migraine*

Women are more often affected than men. The age of onset is usually around puberty, except for women of menopausal age. Migraine tends to become less severe with passing years. The actual attacks may be precipitated by fatigue, anxiety, alcohol, or certain foods, but frequently no reason exists for the individual attack. It is quite common for patients to develop their headache *after*, and not during, a period of stress, the so-called "let down" or "week-end" headache. The duration of attacks is usually of a few hours, sometimes much less, or up to twenty-four hours and very rarely forty-eight hours. The frequency of attacks is varied, but they seldom present more frequently than once or twice a month. Any headache occurring daily or several times a week or which is continuous for days at a time is *not* migraine.

The clinical picture of migraine may be divided into the prodromal or pre-headache stage and the headache phase.

The pre-headache stage of migraine consists of symptoms of diminished cerebral function due to constriction of intracranial arteries. Of these, visual symptoms are the most constant and consist of flashing lights, teichopsia, progressing to a partial or complete homonymous hemianopia. At the same time, paraesthesiae of one side of the body, a mild hemiparesis, or dysphasia may occur. Vertigo is occasionally prominent. Such symptoms are frightening and, whether or not the characteristic unilateral headache of migraine follows, may suggest serious intracranial disease.

Headache is the predominant symptom of migraine and affects the opposite side of the head to the cortical symptoms. It usually starts behind one eye and spreads to involve one half of the head. Vomiting occurs at the height of the headache and will relieve the headache in milder cases. Vomiting may sometimes so overshadow the headache that an acute abdominal disorder may be suspected. These cases of acute episodic gastro-intestinal symptoms may be called "abdominal migraine" and a family history of other episodic disorders such as asthma and hay fever or epilepsy may be obtained.

Ophthalmoplegic migraine is a condition in which recurrent unilateral vascular headache is associated with transient ocular nerve palsies. Although investigation may occasionally show no abnormality a cerebral aneurysm or tumour will be found in most cases.

### *Treatment of Migraine*

The treatment of migraine could be summarized in the words "Ergotamine tartrate and the personality of the physician"!

The importance of ergotamine tartrate lies in its specific vaso-constrictor effect on extracranial arteries. If given in sufficient dosage at an early stage of the migrainous attack, it will nearly always ameliorate the severity of the symptoms. It may be given by mouth, by suppository or by injection. Oral tablets and suppositories usually contain 1–2 mgm ergotamine tartrate, perhaps with the addition of caffeine and simple analgesics. For each attack three or four tablets or suppositories may be used. Maximum effect is obtained by the subcutaneous injection of not more than 0·5 mgm ergotamine tartrate. It must be remembered that large does of ergotamine, however given, may produce vomiting. In mild cases simple analgesics may suffice to relieve headache. Diuretics can be used for those patients who show fluid retention especially in the pre-menstrual phase. When attacks become more frequent or more prolonged reassurance and sedation is needed to allay the inevitable tension anxiety state which is present.

Surgery has no place in the treatment of migraine.

### PERIODIC MIGRAINOUS NEURALGIA

This is a remarkable condition in which episodes of intense unilateral headache lasting from half-an-hour to several hours occur with striking regularity, mainly at night, over a period of a few weeks. The patient feels in normal health between the attacks. The cluster of headaches may continue for a few weeks with intervals of freedom for months or years.

A man of fifty-one complained of attacks of head pain in 1949, 1954 and 1959. Each attack lasted about a month. During this time, he suffered attacks of excruciating left temporal pain, rising within one or two minutes of the onset to their height, and lasting from half to three-quarters of an hour. The attacks usually occurred at night and sometimes by day and were of such regularity that he "could set his watch by them". The pain died away as quickly as it came. During the attack his left eye watered and the left nostril was blocked. There was some reddening and sweating on the left side of the face and the left temporal artery became prominent. The headache was of burning, throbbing or bursting quality and he felt as though he wished to beat his head against a wall. After the attack he felt exhausted for a few minutes, and was then entirely normal. There was no nausea or vomiting. In between these "cluster" attacks he felt entirely normal and never suffered from headaches.

Ergotamine tartrate or di-hydro-codeine by injection usually abort the individual attack. Symonds (1952) has suggested daily injections of ergotamine tartrate 0·25 mgm during the expected duration of the attack and has claimed good results in the prevention of attacks by this means.

### TEMPORAL OR GIANT CELL ARTERITIS

Temporal or giant cell arteritis is uncommon but not rare. It is a disease of the elderly. Although sometimes called "temporal" arteritis, it is a *generalized* sub-acute inflammation which may affect any artery in the body.

Headache is often preceded by a stage of malaise, anorexia, weight loss, fever and sweating, which may continue throughout the illness. The site of the headache is related to the vessels of the scalp which are affected. Temporal or occipital arteries may both be involved and the inflammatory process may start in one vessel and circle the head. During the active stage, the vessels are thickened and prominent with overlying erythema or oedema. As the inflammatory process burns itself out, the artery undergoes thrombosis, finally to change into a string-like cord under the skin. At the height of the illness or later, the patient may be struck by sudden blindness in one or both eyes; this is permanent. Thrombosis of other major arteries may more rarely occur, but this stage should nowadays seldom be reached as treatment with steroids will usually control the inflammatory process. If only one artery is involved, the pain may be instantly relieved by stripping the sympathetic coat surrounding the vessel or by excision of a segment of the artery and its coverings. This, of course, does not attack the underlying process nor prevent involvement of other more important arteries.

### HEADACHES DUE TO CHANGES IN CEREBRO-SPINAL HYDRODYNAMICS

Changes of pressure within the cranial cavity may produce headache. Both low and high C.S.F. pressures are believed to cause symptoms by traction on pain-sensitive intracranial structures (see table) although both may exist in the absence of symptoms.

With high C.S.F. pressure from an expanding or obstructive intracranial lesion the headache is most prominent in the morning and gradually lessens during the day. It is deep and aching but seldom of great severity except in the later stages and often largely relieved by simple analgesics. It is aggravated by coughing and straining. Vomiting may occur at the height of the headache and is less frequently preceded by nausea than other varieties of vomiting. Temporary syncope or blindness may result from bending or straining.

The major characteristic of low pressure headache is that the headache is at its worst when the patient is in the erect posture and is immediately relieved by lying flat or with the head down. Other symptoms of the low pressure state include vertigo, vomiting, syncope and even coma and death. The commonest example of a low pressure state is that following lumbar puncture. If this should develop despite the use of a small needle, the patient should return to bed, with the foot of the bed raised, for twenty-four to forty-eight hours, and the fluid intake must be increased.

A woman of twenty-four was admitted to the Middlesex Hospital having a month before developed hysterical retention of urine for which she had restricted her daily intake of fluid to one pint and had been catheterized once daily in the casualty departments of various London hospitals. A lumbar puncture was performed and a low pressure recorded; fluid was obtained with difficulty. On the following day she was placed on a bed-pan and a few moments later was heard to collapse. On examination, she was found to be deeply comatose with widely dilated, unresponsive pupils, absent deep reflexes and extensor plantar responses. A diagnosis of intracranial hypotension was suggested and she was treated with intravenous fluid; within twenty-four hours she had regained normal consciousness.

It seems likely that this girl would have died had the correct diagnosis and treatment not been established. In such severe cases symptoms can rapidly be dispersed by the intrathecal injection of sterile water.

### REFERRED HEADACHE

*The eye.* Nearly all patients with headache have their eyes tested and yet the headache often remains. Although pain in or around the eye is extremely common it rarely arises from noxious stimuli of the eye, extra-ocular muscles, or the optic nerve. Refractive errors may cause headache as a result of sustained contraction of the extra- or intra-ocular muscles or of the scalp muscles. These headaches may be relieved by proper lenses or re-alignment of the visual axes. A rise of intra-ocular pressure as in glaucoma often produces unilateral headache.

*The nose.* Many patients say that they suffer from "sinus" headaches. Conversely, extensive nasal and paranasal disease may be present without headache. It has been demonstrated that the most pain-sensitive nasal structure is the mucosa lining the ostia of the para-nasal sinuses. The mucous lining of the sinuses is of relatively low sensitivity. It may therefore be said that if a headache is not associated with inflammation or engorgement of the turbinates, the headache is unlikely to be the result of disease of nasal structures. If, however, the pain is relieved by anaesthetizing these structures then the headache probably has a nasal origin.

Malignant disease of the paranasal structures may produce pain by invasion of bone or nerves.

Infected teeth and an unstable temporo-mandibular joint, the so-called Costen's syndrome, may also produce head pain. But in all varieties of referred headache, the local symptoms and signs usually overshadow the head pain to which is frequently added the element of muscle contraction.

### INTRA-CRANIAL HAEMORRHAGE

The headache of massive intracerebral or subarachnoid bleeding demands no description; it is catastrophic and overwhelming but soon immersed in the depths of unconsciousness. Lesser degrees of subarachnoid bleeding may pass unnoticed, unless the diagnosis is considered in any case of sudden headache, especially if the patient is young. Signs of meningeal irritation should be sought, and if they are present, lumbar puncture is indicated.

Extradural haemorrhage is the result of rupture of the branches of the middle meningeal artery. The blow that causes this may not necessarily be severe, although the patient is usually temporarily concussed. In some cases there follows a lucid interval in which normal activities may be continued. After a few hours, during which headache is increasingly prominent, there is progressive drowsiness leading to coma and death. This constitutes a neurosurgical emergency.

In subdural haemorrhage the sequence of events is elongated and protracted as compared with extradural haemorrhage. The blow to the skull may also be trivial and the patient may or may not be dazed or concussed. The latent interval before headache starts is of days, more often weeks, or occasionally months. The headache, drowsiness and intellectual lethargy are curiously fluctuating both during the day and from day to day, but the progress is progressively downhill until the coma is deep and the patient succumbs. Until this later stage of the illness the mental changes greatly overshadow the neurological signs which comprise some degree of papilloedema, hemiparesis, sometimes on the same side as the haematoma, and slight neck stiffness. The C.S.F. may be xanthochromic and may contain an excess of protein, lymphocytes and red cells. Early evacuation of the clot is essential and the neurosurgeon must always bear in mind that subdural haematoma may be bilateral.

### POST-TRAUMATIC HEADACHE

This is relatively common when the thought of compensation exists.

During World War II the experiment of rapid mobilization and rehabilitation of patients with severe head injuries was tried with remarkable success. This approach is at variance with the policy adopted by some clinicians and many of the laity may find such a policy difficult to understand, especially as there is a general belief that permanent brain damage may follow such injuries. This point of view may be strengthened if members of our profession tell patients that they must *expect* to have headache after severe head injury. This is rarely true.

However, headache frequently does occur after head injury. It has the characteristics of muscle contraction or tension headache, and carries with it many other anxiety symptoms. The perpetuating force of these headaches may be the fear of organic brain damage because the head has a special symbolic significance as the centre of thought, memory and learning. Many of the patients who develop post-traumatic headache have displayed neurotic traits before the accident and are of poor emotional fibre whilst a very few are true malingerers.

### TENSION HEADACHE

The word "tension" may have two meanings. It may mean that sustained contraction or tension of the scalp muscles is the cause of this type of headache, or that nervous tension or strain is the underlying cause. Sustained contraction of neck and scalp muscles can be demonstrated by electro-myography. Like any other muscle cramp, this ache in the occipital muscles may be reduced by gentle movement and stretching, and exaggerated by repeated powerful movement. Heat and massage ease the spasm and relieve the pain temporarily. Tension headache may follow as a consequence of any other head pain especially when the patient is anxious or tense. It is the cause of the daily or prolonged headaches which occur in migraine. It is the major cause of the headache seen in cervical spondylosis and may overshadow the headache of a cerebral tumour.

The symptoms are variable and sometimes bizarre. Headache is seldom the only symptom and is usually accompanied by such symptoms as excessive tiredness, depression, irritability, failing memory and concentration, trembling, spots before the eyes and many others. The head pain may affect any part of the head or neck and is often described as a heavy weight, like a vice or a tight band, pressure, stiffness or a drawing sensation. Patients often notice that the scalp is tender when the hair is brushed. Tension headaches last for hours, days or weeks, waxing and waning in severity but never ceasing entirely. They are provoked by periods of stress and anxiety and are especially likely when no solution can be found to the immediate problem. "Let down" tension headache occurs, as in migraine.

*The Management of Tension Headaches*

It is essential that the patient should appreciate that his doctor is interested in his problems and understands his symptoms. These are very real to the patient who is unlikely to be reassured by cursory anamnesis and examination. The patient must be asked to discuss any source of worry or stress, however trivial it may seem. Examination must then be detailed and it is always wise to pay attention to the head and neck although no more than some tenderness is to be expected.

An X-ray of the skull is always reassuring to the patient although rarely informative to the doctor and should be done in patients whose headaches persist despite treatment. If the clinician has any suspicion of organic disease, an E.E.G. should also be done.

When the clinician is certain that the problem with which he deals is entirely non-organic, he must give the whole weight of his reassurance to the patient together with some simple explanation of how muscle spasm produces discomfort or pain. It is of no value to tell the patient that there is nothing the matter with him and that it is all due to "nerves" as this only serves to reinforce the patient's view that the doctor does not really understand his particular problem. It is of little value to tell patients "to pull themselves together" unless they know how their symptoms arise and know that the cause is benign.

In many patients simple reassurance is sufficient to dispel symptoms, but in others sedation is necessary. It is illogical to administer heavy night sedation only to leave the patient dull and depressed in the morning and unsedated for the rest of the day. Sedation should cover the whole twenty-four hour period and should be given at twelve- or eight-hourly intervals. The range of sedative drugs now available makes it unlikely that any patient will be intolerant of them all. The drug should be used for some weeks in gradually diminishing dosage and then prophylactically before any especially worrying circumstance.

While gentle physiotherapy is often of great help, it is not logical to manipulate the necks of tense patients and the use of a cervical collar may add yet another mental burden to the patient. The treatment of tension headaches depends on an understanding of the patient's problems, simple psychotherapy and sedation, but at the same time it must be recognized that there are many people who are incapable of facing the problems of the world and who may develop a more dire symptom should the headache be relieved. In such circumstances headache is the best compromise that can be made with life.

REFERENCES

Brain, Russell. (1955). *Diseases of the Nervous System* (Oxford University Press).
Symonds, C. (1952). *Trans. med. Soc. Lond.* (1950–1951), **67**, 237.

